# Daytime Central Thalamic Deep Brain Stimulation Modulates Sleep Dynamics in the Severely Injured Brain: Mechanistic Insights and a Novel Framework for Alpha-Delta Sleep Generation

**DOI:** 10.3389/fneur.2019.00020

**Published:** 2019-02-04

**Authors:** Jackie L. Gottshall, Zoe M. Adams, Peter B. Forgacs, Nicholas D. Schiff

**Affiliations:** ^1^Feil Family Brain and Mind Research Institute, Weill Cornell Medicine, New York, NY, United States; ^2^Department of Neurology, Weill Cornell Medicine, New York, NY, United States; ^3^Rockefeller University Hospital, New York, NY, United States

**Keywords:** traumatic brain injury, minimally conscious state, deep brain stimulation, synaptic homeostasis hypothesis, alpha-delta sleep, sleep spindles, slow wave sleep

## Abstract

Loss of organized sleep electrophysiology is a characteristic finding following severe brain injury. The return of structured elements of sleep architecture has been associated with positive prognosis across injury etiologies, suggesting a role for sleep dynamics as biomarkers of wakeful neuronal circuit function. In a continuing study of one minimally conscious state patient studied over the course of ~8½ years, we sought to investigate whether changes in daytime brain activation induced by central thalamic deep brain stimulation (CT-DBS) influenced sleep electrophysiology. In this patient subject, we previously reported significant improvements in sleep electrophysiology during 5½ years of CT-DBS treatment, including increased sleep spindle frequency and SWS delta power. We now present novel findings that many of these improvements in sleep electrophysiology regress following CT-DBS discontinuation; these regressions in sleep features correlate with a significant decrease in behavioral responsiveness. We also observe the re-emergence of alpha-delta sleep, which had been previously suppressed by daytime CT-DBS in this patient subject. Importantly, CT-DBS was only active during the daytime and has been proposed to mediate recovery of consciousness by driving synaptic activity across frontostriatal systems through the enhancement of thalamocortical output. Accordingly, the improvement of sleep dynamics during daytime CT-DBS and their subsequent regression following CT-DBS discontinuation implicates wakeful synaptic activity as a robust modulator of sleep electrophysiology. We interpret these findings in the context of the “synaptic homeostasis hypothesis,” whereby we propose that daytime upregulation of thalamocortical output in the severely injured brain may facilitate organized frontocortical circuit activation and yield net synaptic potentiation during wakefulness, providing a homeostatic drive that reconstitutes sleep dynamics over time. Furthermore, we consider common large-scale network dynamics across several neuropsychiatric disorders in which alpha-delta sleep has been documented, allowing us to formulate a novel mechanistic framework for alpha-delta sleep generation. We conclude that the bi-directional modulation of sleep electrophysiology by daytime thalamocortical activity in the severely injured brain: (1) emphasizes the cyclical carry-over effects of state-dependent circuit activation on large-scale brain dynamics, and (2) further implicates sleep electrophysiology as a sensitive indicator of wakeful brain activation and covert functional recovery in the severely injured brain.

## Introduction

Increasing evidence suggests that many patients with disorders of consciousness experience neuronal re-organization and recovery of large-scale brain function over prolonged time periods (1–5). The presence of sleep architecture, particularly sleep spindles and slow wave sleep (SWS), has been found to correlate with prognosis following injury and therefore may be an important dimension for understanding and tracking functional improvements (6–14). In the present study we tracked long-term sleep changes in a single patient remaining in minimally conscious state over ~8.5 years. Adams et al. (15) first reported on the EEG sleep characteristics of this patient before and during ~5 years of daytime central thalamic deep brain stimulation (CT-DBS), which was initiated for the promotion of arousal regulation. Initial findings by Adams et al. showed an increase in sleep spindle frequency and SWS duration following the onset of CT-DBS treatment (15). An irregular intrusion of alpha activity during SWS was also reported prior to CT-DBS, which seemed to attenuate during CT-DBS treatment. Importantly, these results implicated daytime brain activation as modulator of sleep architecture in the severely injured brain.

Here we report on distinct changes observed ~1 year after the discontinuation of CT-DBS treatment. We find ongoing plasticity in multiple physiological aspects of sleep, providing important insight into the network-level dynamics that can be induced by daytime arousal regulation in the severely injured brain. Most notably, in the present study we identify a regression of the previously noted improvements in sleep dynamics seen over course of CT-DBS treatment. A return of the atypical SWS alpha intrusion initially reported by Adams et al. as “mixed state” is evaluated here in the context of the previously documented phenomenon “alpha-delta sleep” (16). We discuss our findings in the context of neuronal circuit mechanisms that may organize the improvement of sleep dynamics during daytime CT-DBS in the severely injured brain, as well as those that may underlie functional regression with the withdrawal of CT-DBS treatment. Finally, we explore the role of alpha-delta sleep across pathophysiologies of neuropsychiatric disorders and propose a mechanistic explanation for alpha-delta sleep generation.

## Methods

### Patient History

Patient subject is a 48-year-old man who suffered a severe brain injury as the result of a motor vehicle accident at the age of 17. The injury pattern is characterized by a small left thalamic hemorrhage, as well as diffuse axonal injury with extensive atrophy of the left hemisphere (Supplementary Figure 1). Behavioral presentation has remained consistent with a diagnosis of minimally conscious state since the time of injury.

### Data Collection and Timeline

**Figure 1:**
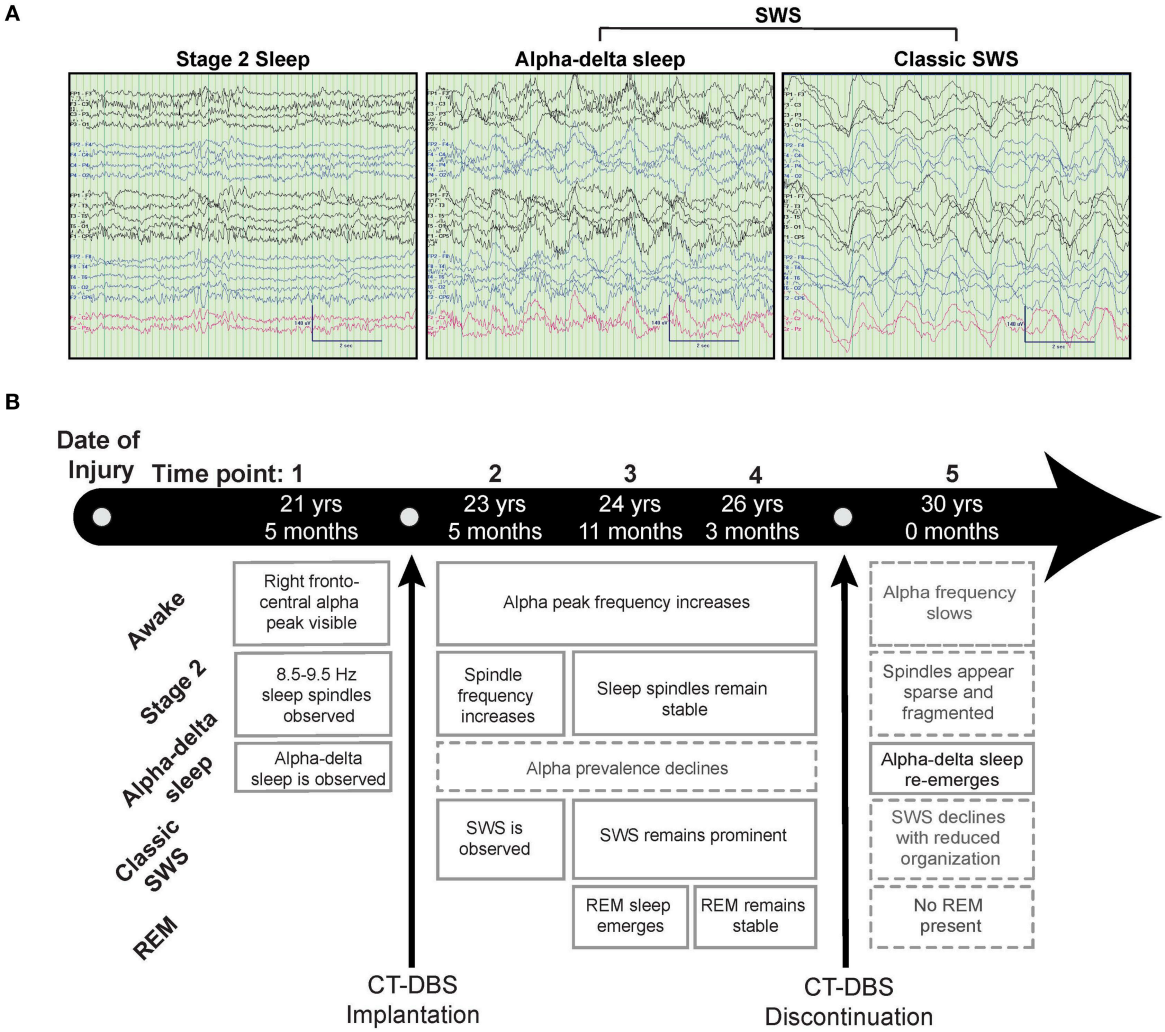
Representative EEG and summary timeline. **(A)** Representative segments of patient subject EEG tracings during non-REM sleep show the presence of stage two (left) and SWS (middle, right) epochs. A mixed frequency signature consistent with alpha-delta sleep was also observed (middle), classified here as a subset of SWS for consistency with combined “SWS-like” states reported in Adams et al. (15). **(B)** The patient subject was studied at five time points over the course of 8.5 years, consisting of one time point before, three during, and one after CT-DBS treatment. Qualitative summaries of individual EEG states are shown for each time point.

The patient subject was studied longitudinally at five time points (TP1-TP5) over the course of 8.5 years (Figure 1). Each study consisted of a 24–72 h inpatient admission (TP1 at New York Presbyterian Hospital, New York, USA; TP2-TP5 at Rockefeller University Hospital, New York, USA) under IRB approvals from Weill Cornell Medicine and Rockefeller University. Written informed consent was obtained from the patient's surrogate for study participation, data collection and publication. During each inpatient study, behavioral responsiveness was quantified according to the Coma Recovery Scale-Revised (CRS-R) (17) at least once per day. Overnight video-EEG was collected with collodion-pasted electrodes (30 electrodes at TP1; 37 electrodes at TP2-TP5) placed according to the international 10–20 system. Signals were recorded using the Natus XLTEK system (San Carlos, CA) at impedances ≤ 5 kΩ. Time point one occurred at 21 years 5 months post injury. The patient subject subsequently underwent surgery for the implantation of bilateral central thalamic deep brain stimulation electrodes [clinical trial methods are described briefly below, as well as in detail in Schiff et al. (18)]. Study time points two through four were concurrent with daytime central thalamic deep brain stimulation (CT-DBS) (TP2: 23 years 5 months, TP3: 24 years 11 months, and TP4: 26 years 3 months post injury, respectively). Time point five occurred ~1 year following CT-DBS discontinuation secondary to battery depletion (TP5: 30 years 0 months post injury).

Implantation of deep brain stimulation electrode leads was aided by microelectrode recordings from the sensory relay nucleus of the thalamus on both the right and left hemispheres. Using electrophysiological localization of the sensory nucleus as known, the lateral wing of the central lateral nucleus was targeted using the Morel atlas (19) by dead reckoning. Following implantation, CT-DBS was administered on a blocked 12 h ON/OFF cycle (ON: 6AM-6PM/OFF: 6PM-6AM) over the course of ~7 ½ years. During this time, a wide range of electrode contact geometries, stimulation intensities, and frequencies of stimulation were employed. Briefly, following extensive titration testing of stimulation frequency and intensity, an optimal geometry was identified for each electrode contact based on apparent arousal effects and limitations of visible side effects. The left electrode was stimulated in bipolar mode with the lowest contact chosen as cathode and highest contact chosen as anode; the right electrode was stimulated in monopolar mode with two cathodes, placed at the lowest two contacts. During a 6 month crossover phase, stimulation at these contacts occurred for 12 h each day using a 90 ms pulse width, 130Hz stimulation frequency, and 4 V intensity for each electrode. Following the crossover phase, a range of varying frequencies and intensities were used including a 1 year period of stimulation at each of 175Hz and 100Hz. For the majority of the 7½ years of CT-DBS exposure stimulation occurred at 100Hz with other parameters held constant.

### Data Analysis

#### EEG Processing

For estimates of wakeful brain dynamics, periods of resting eyes open awake states were identified by video record and corresponding EEG was manually cleaned for the removal of eye blink and movement artifacts.

Sleep analyses included nighttime sleep EEG collected between the hours of 8 p.m. and 6 a.m., manually cleaned for movement artifacts and verified eyes-closed according to synchronous video record. Standard sleep scoring criteria were used to classify segments as stage two or SWS (20). Briefly, the patient subject was considered to be in stage two sleep if the EEG record displayed k-complexes and/or 9–16Hz spindle-like formations across frontocentral channels. Slow wave sleep was classified by large polymorphic delta (<4Hz) waves present over at least 20% of a 30 s epoch. The observation of an additional and constant 8–14Hz oscillation overriding classic SWS characteristics was considered alpha-delta sleep. To maintain consistency with the previous report by Adams et al. (15), alpha-delta sleep was scored under the categorization of SWS.

#### Power Spectral Estimation

Raw EEG was segmented into 30–35 representative epochs for awake, stage two sleep, and SWS states, respectively. Multitaper power spectral estimates were calculated separately for each state (21) with implementation of Hjorth Laplacian montaging from the MATLAB chronux toolbox (22). After spectral calculation, six frontocentral channels (F3, F4, FC5, FC6, C3, C4) were used for longitudinal comparison to maintain consistency with Adams et al. (15).

For longitudinal comparisons of spectral peak sizes during stage two and SWS stages, calculated spectra were normalized according to methods outlined in Gottselig et al. (23). Briefly, a power law function was fit to each spectrum in the 5–6Hz and 17–18Hz frequency ranges for stage two sleep, or 4–6Hz and 23–24Hz frequency ranges for SWS. Frequency ranges for normalization were chosen based on optimal fitting of the power law function to the underlying shape of the power spectrum across channels, excluding frequency bins of interest to avoid flattening of relevant spectral features. Absolute power of the fitted spectrum was subtracted from the calculated spectrum and resulting values were integrated across frequency bins of interest. By subtracting the best-fit underlying spectral shape, arbitrary differences in background power bias between visits were removed, allowing for estimation of magnitude change in relevant spectral features.

Dominant spindle frequency was determined from normalized stage two power spectra using a handcrafted manual click program to determine the center frequency of the largest spectral peak in the 9–16Hz spindle range. Briefly, the spindle peak for each channel was visually identified from the normalized power spectrum and a quadratic polynomial was fit to the identified peak to determine the local power maxima and corresponding dominant spindle frequency. If no spindle peak was present no value was recorded.

#### Statistics

Analyzed variables were CRS-R total score, stage two sleep spindle power (9–16Hz) and peak spindle frequency, SWS delta power (0.5–4Hz), and SWS alpha power (8–14Hz). Time periods for comparison were grouped into three conditions reflecting initial pre-stimulation baseline, the active period of CT-DBS, and the post-withdrawal of stimulation phase (Pre:TP1/Active:TP2-TP4/Post:TP5). An analysis of variance (ANOVA) was performed for each variable to identify changes across CT-DBS conditions. For stage two and SWS variables, ANOVA factors included CT-DBS condition and hemisphere. To identify any changes within the active CT-DBS condition, a separate ANOVA was conducted for TP2-TP4 within each variable. *Post-hoc* comparisons were conducted using Tukey's HSD at a significance level of *p* < 0.05.

## Results

### Visual EEG Features

Figure 1 provides a qualitative summary of changes in EEG architecture over the course of study. Most notable was the observation at TP1 of an additional sleep signature consisting of high voltage, low frequency (<2Hz) activity exhibiting an overriding mid-frequency (8–14Hz) component (Figure 1A, middle panel). This signature closely resembles alpha-delta sleep, characterized by Hauri and Hawkins (16) as “a mixture of 5–20% delta waves (>75 μV, 0.5–2 c/sec) combined with relatively large amplitude, alpha-like rhythms (7–10 c/s).” Alpha-delta sleep was prominent before CT-DBS treatment (TP1), waned during active CT-DBS (TP2-TP4), and re-emerged following discontinuation of CT-DBS (TP5). Inversely, changes in healthy sleep architecture during CT-DBS treatment included the normalization of stage two sleep spindles, SWS, and awake alpha rhythms, as well as the emergence of REM sleep. Each of these healthy features demonstrated qualitative decline following CT-DBS discontinuation (Figure 1B).

### Behavioral Examination

**Figure 2:**
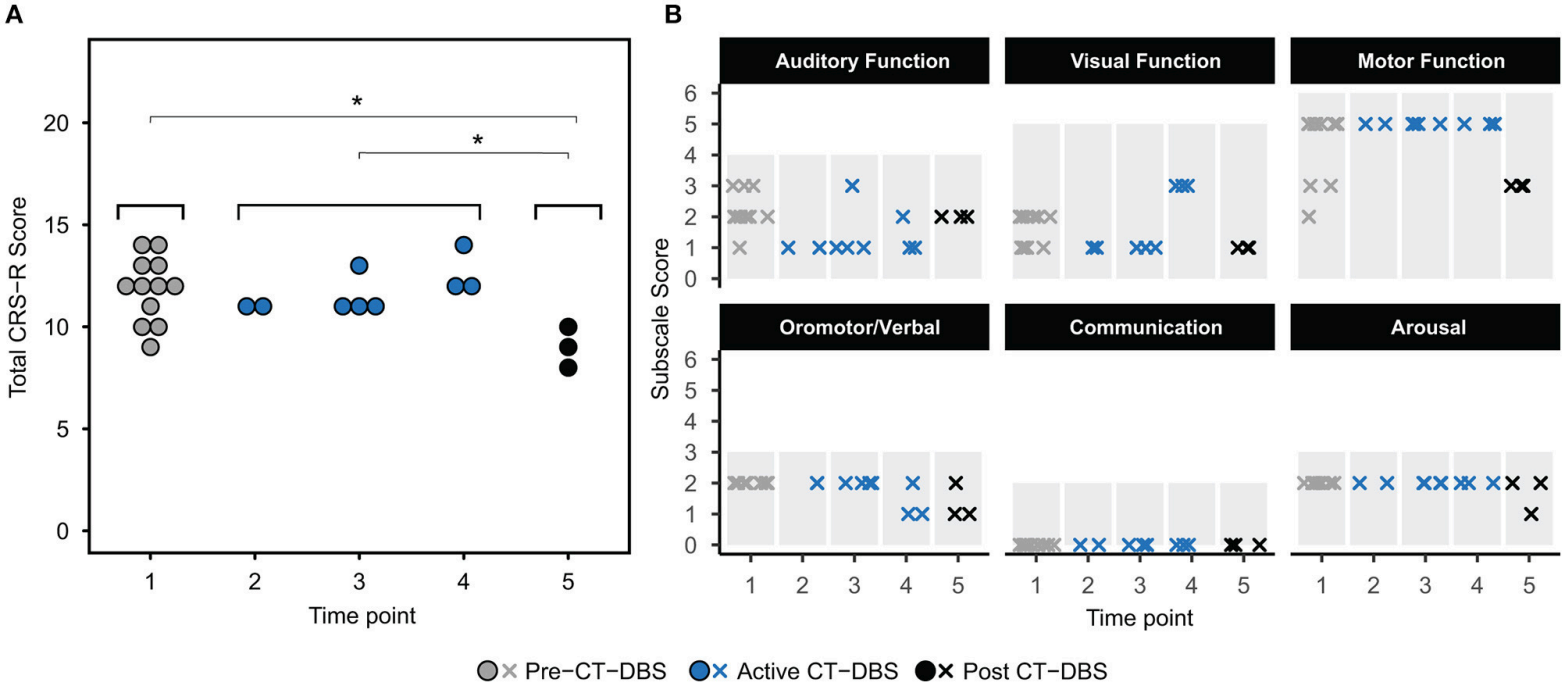
Behavioral examination scores. **(A)** Coma Recovery Scale-Revised (CRS-R) total scores at each time point. Each data point represents a single CRS-R administration. ^*^p < 0.05. **(B)** Corresponding CRS-R subscale scores display slight variations in composition of total CRS-R scores between time points. Each data point represents a single subscale administration. Gray rectangles indicate maximum subscale score range. Data points from pre-CT-DBS are shown in gray, active CT-DBS in blue, and post-CT-DBS in black.

The CRS-R was administered at least once daily during each time point. A one-way ANOVA showed a significant effect of CT-DBS condition (pre, active, post) [*F*_(2, 21)_ = 5.55, *p* = 0.0116], such that total CRS-R scores were significantly lower after CT-DBS cessation (*M* = 9.0, *SD* = 1.0) than either before CT-DBS (*M* = 11.8, *SD*=1.6, *p*=0.011) or during active CT-DBS (*M*=11.8, *SD* = 1.1, *p* = 0.016) (Figure 2A). Although this reduction in CRS-R score was statistically significant, the patient subject remained within the diagnostic classification of minimally conscious state throughout the course of study. There was no change in CRS-R scores between active CT-DBS time points.

CRS-R subscale scores were also compared for a detailed view of composite CRS-R score changes. Analysis of variance was not performed due to the categorical nature of subscale classifications. Subscale scores varied slightly across time points, with the exception of the communication subscale, for which the patient subject received a score of 0 at each examination (Figure 2B). Altogether, although CT-DBS did not produce an increase in CRS-R scores, the withdrawal of CT-DBS correlated with a significant reduction in responsiveness at TP5.

### Power Spectra During Wake, Stage 2, and SWS

**Figure 3:**
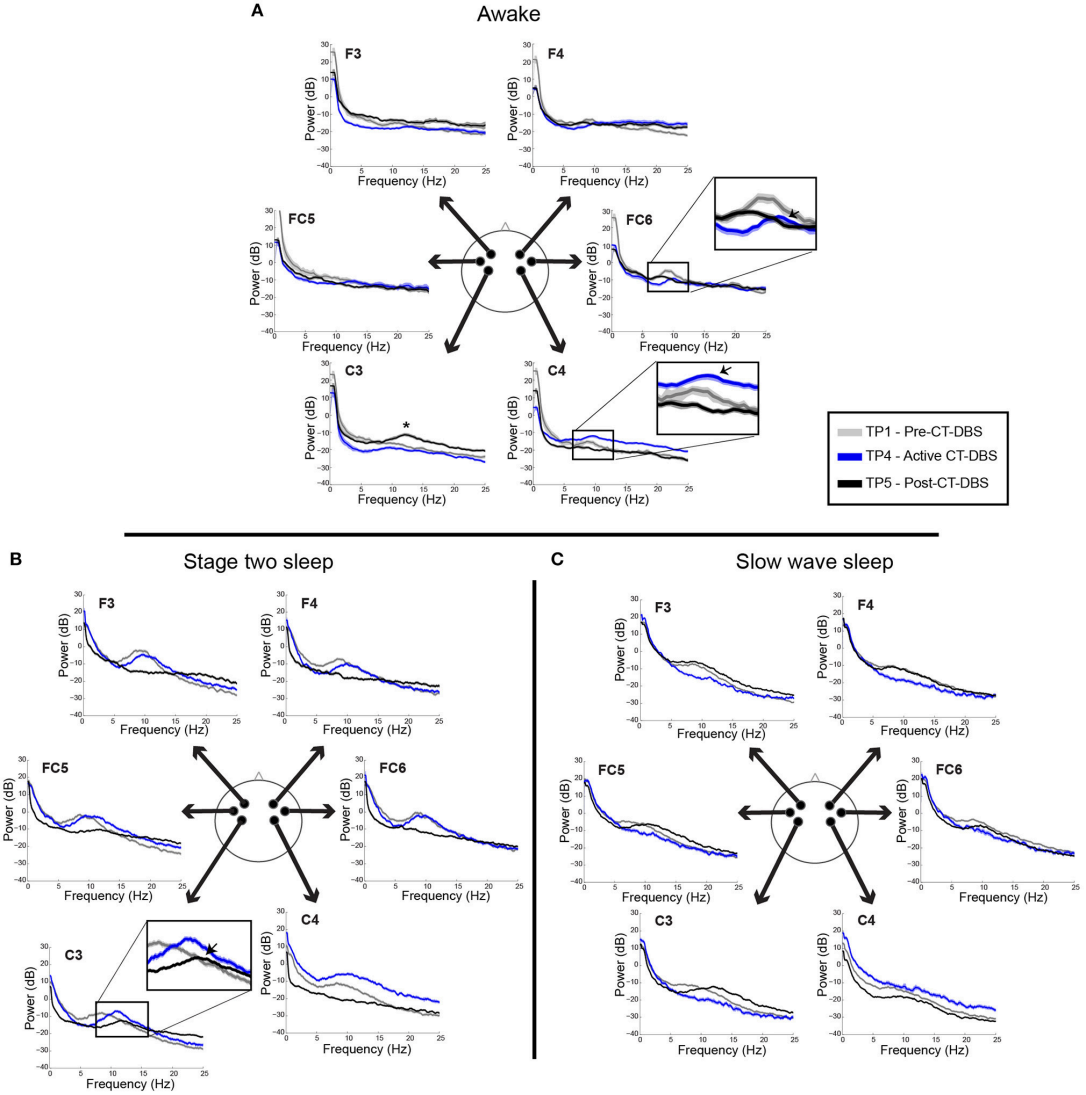
EEG power spectra from time points 1, 4, and 5, corresponding to pre-, active, and post-CT-DBS conditions. **(A)** Power spectra calculated from resting awake EEG. ^*^ Indicates emergence of a “wicket rhythm” (~8–13Hz) over the left motor cortex. FC6 and C4 inset arrows denote increases in peak alpha frequency at time point 4. **(B,C)** Power spectra calculated from non-REM stage two sleep **(B)** and SWS **(C)**. Spectral tracings from time point 1 (TP1) are represented by gray lines, time point 4 (TP4) by blue lines, and time point 5 (TP5) by black lines.

Power spectra from TP1, TP4, and TP5 were overlaid for a qualitative analysis of spectral shape before, during, and after CT-DBS, respectively. Awake power spectra showed small local changes but few global changes over time (Figure 3A). In the alpha range, FC6 initially demonstrated a spectral peak at ~8–9Hz which reduced in power but increased in frequency to ~9–10Hz by TP4 (Figure 3A, FC6 inset arrow). Following discontinuation of CT-DBS at TP5, the FC6 power spectrum largely flattened and showed no clear peak within the alpha range (Figure 3A, FC6 inset). A similar awake alpha modulation was present in C4 with a less prominent increase in alpha frequency from ~8Hz at TP1 to ~9Hz at TP4 (Figure 3A, C4 inset arrow) and a complete flattening at TP5 (Figure 3A, C4 inset). Additional examination of parietal and occipital channels during wakeful periods also revealed increases in alpha frequency at TP4 with slight reductions at TP5 (data not shown). Channel C3 uniquely showed prominent electrophysiological change after CT-DBS was discontinued with the emergence of a clear spectral peak in the beta frequency range at ~12Hz during TP5 (Figure 3A, C3, asterisk).

In contrast to the variable results observed in the patient subject's awake EEG, spectral analysis of stage two sleep showed robust global changes over time. Power spectra were characterized by an increased peak frequency in the sleep spindle range from TP1 to TP4 across all channels (Figure 3B). Following CT-DBS discontinuation at TP5, power in the spindle range disappeared entirely in all channels except C3. At TP5, C3 showed a continued increase in peak spindle frequency, albeit displaying a smaller and less defined spectral peak (Figure 3B, C3 inset arrow). These findings are consistent with the observation of sleep spindle fragmentation across the majority of EEG channels following CT-DBS discontinuation.

SWS power spectra also demonstrated global changes over time, most notably characterized by an intrusion of 8–14Hz power across channels prior to CT-DBS treatment at TP1, corresponding to the presence of alpha-delta sleep (Figure 3C). The 8–14Hz alpha-delta sleep frequency signature was absent in all channels during active CT-DBS treatment at TP4, only to re-emerge following CT-DBS discontinuation at TP5. Re-emergence of alpha-delta sleep at TP5 showed increased peak frequency in the alpha range across all except for the two frontal channels (F3 and F4).

### Relationship Between Sleep Dynamics and CT-DBS

For statistical comparison, data were collapsed into three groups: “Pre CT-DBS” (TP1), “Active CT-DBS” (TP2-TP4), and “Post CT-DBS” (TP5). Global feature measurements were compared across and within CT-DBS conditions for a quantitative analysis of the effects of CT-DBS treatment and subsequent cessation on EEG sleep dynamics.

#### Stage Two Sleep Spindles

**Figure 4:**
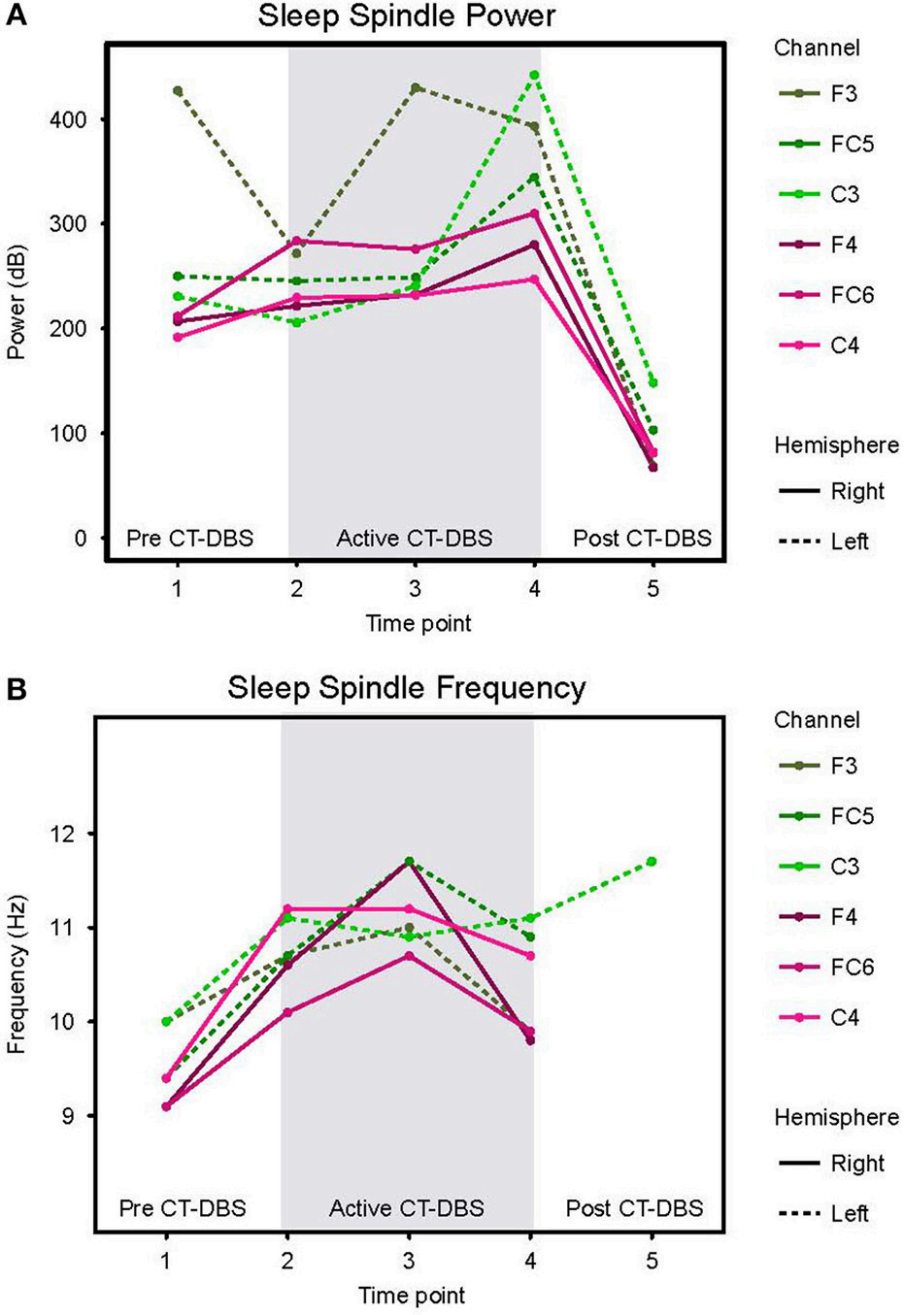
Stage two sleep spindle dynamics. Power spectra were calculated for representative stage two sleep EEG segments and values normalized for comparison across time points. **(A)** Power in the 9–16Hz spindle range, plotted according to channel and time point. **(B)** Peak spectral frequency in the 9–16Hz spindle range. If no spectral peak was present, spindles were considered absent and no frequency value was recorded. Dotted lines represent left hemisphere channels and solid lines represent right hemisphere channels. Individual channels are plotted by color. Gray shading indicates active CT-DBS time points.

Normalized stage two power spectral calculations were used to quantify changes in spindle (9–16Hz) power over time. A two-way ANOVA with factors CT-DBS (pre, active, post) and hemisphere (left, right) showed greater spindle power in the left hemisphere [*F*_(1)_ = 6.483, *p* = 0.0177], as well as a highly significant main effect of CT-DBS condition [*F*_(2)_ = 20.411, *p* < 0.0001] (Figure 4A, Table 1). *Post-hoc* tests identified a reduction in spindle power post CT-DBS compared to both pre and active CT-DBS conditions, *p* < 0.001 and *p* < 0.0001, respectively. Spindle power remained consistent across the active CT-DBS condition, with the exception of a slight increase in the left hemisphere at TP4 compared to TP2, *p* = 0.0438.

**Table 1:** Average sleep variables calculated from EEG power spectral estimates.

	**CT-DBS condition**	**Stage two spindle power (dB)**	**Stage two spindle frequency (Hz)**	**SWS delta power (dB)**	**SWS alpha power (dB)**
Left Hemisphere	Pre	302.62 ± 108.32	9.80 ± 0.35	23.26 ± 2.08	232.04 ± 17.08
	Active	313.61 ± 90.04	10.89 ± 0.48	104.22 ± 83.29	100.82 ± 18.17
	Post	106.76 ± 39.86	11.70^*^	45.30 ± 23.35	293.66 ± 45.37
Right Hemisphere	Pre	203.35 ± 10.64	9.20 ± 0.17	27.30 ± 3.75	184.90 ± 7.74
	Active	256.80 ± 31.20	10.66 ± 0.64	91.39 ± 63.70	98.10 ± 28.03
	Post	76.91 ± 8.20	NA	46.10 ± 23.35	216.06 ± 25.62

* *Channel C3 only*.

To quantify changes in spindle frequency, we first removed the post CT-DBS condition (TP5) from analyses due to lack of spectral peak in the spindle range in five of the six channels (see Figure 3B). A two-way ANOVA with factors CT-DBS (pre, active) and hemisphere (left, right) demonstrated significantly faster spindle frequency in both hemispheres during active CT-DBS than before CT-DBS treatment [*F*_(1)_ = 26.920, *p* < 0.0001] (Figure 4B, Table 1). Spindle frequency varied within active CT-DBS time points [*F*_(2)_ = 4.158, *p* = 0.0425], such that there was a significant slowing from TP3 to TP4, *p* = 0.0347, with frequency at TP4 consistent with peak spindle frequency at TP2.

#### SWS Delta Power

**Figure 5:**
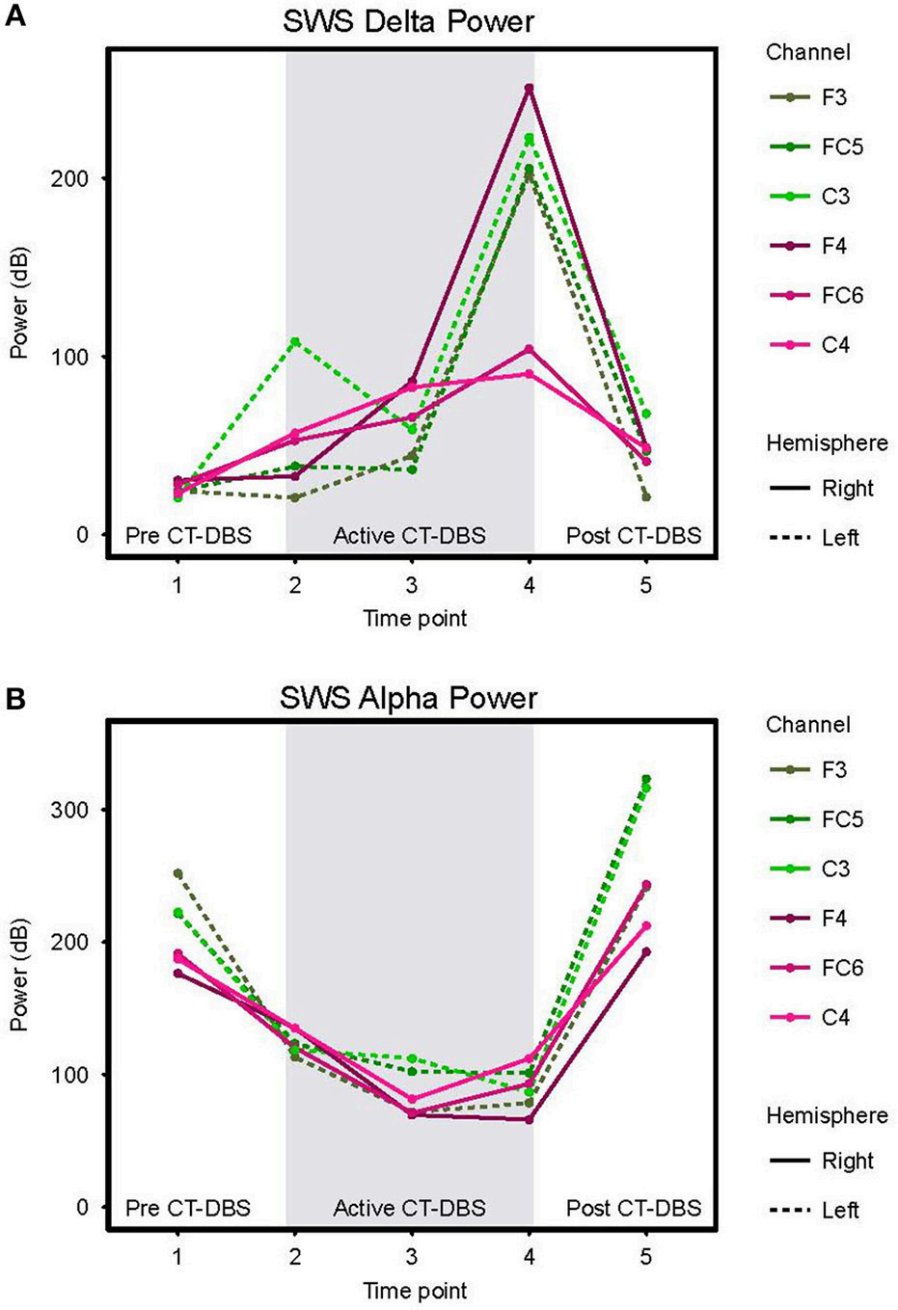
Slow wave sleep dynamics. Power spectra were calculated for representative SWS segments (including alpha-delta sleep) and values normalized for comparison across time points. **(A)** SWS power in the 0.5–4Hz delta frequency range, plotted according to channel and time point. **(B)** SWS power in the 8–14Hz alpha frequency range, plotted according to channel and time point. Dotted lines represent left hemisphere channels and solid lines represent right hemisphere channels. Individual channels are plotted by color. Gray shading indicates active CT-DBS time points.

Delta (0.5–4Hz) power was quantified from normalized SWS power spectra as an indicator of healthy SWS electrophysiology. A two-way ANOVA with factors CT-DBS (pre, active, post) and hemisphere (left, right) showed a global effect of CT-DBS [*F*_(2)_ = 3.932, *p* = 0.033] but not hemisphere, such that SWS delta power significantly increased from pre to active CT-DBS conditions, *p*=0.0472 (Figure 5A, Table 1). During active CT-DBS, delta power was significantly greater at TP4 than TP2 and TP3, *p* < 0.001 and *p* = 0.001, respectively. Despite an empirical reduction in delta power from TP4 to TP5, statistical analyses yielded no difference between active and post CT-DBS conditions, *p* = 0.186.

#### SWS Alpha Power

Alpha (8–14Hz) power during SWS was quantified as a marker of alpha-delta sleep expression. A two-way ANOVA with factors CT-DBS (pre, active, post) and hemisphere (left, right) yielded a highly significant CT-DBS by hemisphere interaction [*F*_(2)_ = 5.657, *p* = 0.00971] (Figure 5B, Table 1). *Post hoc* testing showed that global alpha power was reduced from pre to active CT-DBS conditions, *p* < 0.001, followed by a significant rebound from active to post CT-DBS conditions, *p* < 0.001. This rebound was larger in the left than the right hemisphere, such that SWS alpha power did not differ between hemispheres during pre or active CT-DBS conditions, but was greater in the left hemisphere following CT-DBS withdrawal, *p* = 0.010. SWS alpha power also differed within active CT-DBS time points [*F*_(2)_ = 13.329, *p* < 0.001], such that it was significantly reduced from TP2 to TP3, *p* = 0.001, and remained suppressed at TP4 compared to TP2, *p* = 0.003.

## Discussion

In this longitudinal study of a single patient subject in chronic minimally conscious state, we report marked regression of sleep dynamics following the discontinuation of CT-DBS. In this patient subject, Adams et al. (15) previously demonstrated that daytime CT-DBS (6 a.m.−6 p.m.) over a 5 year period was associated with the normalization of sleep architecture and dynamics; specific changes included increased spindle frequency during stage two sleep, increased sustained SWS, and the re-emergence of REM sleep. Presently, we show regression of each of these improvements observed at 1 year after CT-DBS discontinuation (see Figure 1 for a schematic summary) and in temporal correlation with a significant reduction in behavioral responsiveness (Figure 2). During stage two sleep we identify a loss of sleep spindles alongside a reduction in spectral power in the spindle range (Figures 3B, 4). We also find a reversion of SWS delta power to pre-CT-DBS levels (Figures 3C, 5A), and no instances of REM sleep. Importantly, we observe the re-emergence of a SWS-like frequency signature that had previously been suppressed by daytime CT-DBS (Figure 5B). This frequency signature closely resembles the “alpha-delta sleep” pattern that has been identified across several neuropsychiatric conditions (16, 24–29), leading us to re-characterize this phenomenon as alpha-delta sleep arising within the severely injured brain.

In summary, reduced behavioral responsiveness after CT-DBS discontinuation was associated with the abolishment of stage two sleep spindles, marked downregulation of SWS delta power, and the return of alpha-delta sleep. In the following sections we discuss: (1) the proposed mechanism of sleep modulation by daytime CT-DBS in the severely injured brain and implications for sleep dynamics as an indicator of wakeful engagement, (2) altered network dynamics that may underlie alpha-delta sleep expression in the severely injured brain, and (3) a novel mechanistic framework for alpha-delta sleep generation across pathophysiologies.

### Restoration of Frontostriatal Activation by CT-DBS May Drive Sleep Changes in the Severely Injured Brain

The rationale for using CT-DBS in minimally conscious state patients is two-fold: (1) the central thalamus has widespread innervation of frontal cortical and basal ganglia regions and plays a crucial role in maintaining arousal regulation during wakeful states, (2) multi-focal deafferentation is a characteristic injury pattern in severe brain injuries and is known to functionally and structurally disfacilitate central thalamic neuronal populations (30–32). The upregulation of central thalamic neurons via CT-DBS is therefore expected to re-establish frontostriatal neuronal firing rates, thereby restoring the frontocortical regulation of sustained waking arousal needed to support organized behavior (33). Studies of CT-DBS in another post-traumatic minimally conscious state patient provided proof-of-concept that restoration of sustained frontocortical activity correlates with improvements in organized behavior. In these studies, increased neuronal activity in the frontal cortices produced by CT-DBS was associated with heightened arousal, recovery of speech, restored executive motor control over one limb, and improved feeding behaviors (18, 34). While CT-DBS in our patient subject failed to produce clinically measurable behavioral improvement, it did produce robust improvements in sleep electrophysiology. Adams et al. (15) proposed that these sustained changes in network dynamics visible during sleep were the result of daytime activation of frontostriatal systems by CT-DBS, which allowed for organized neuronal activity in intact but functionally downregulated frontostriatal networks (30, 33, 35–37).

Here we show that CT-DBS cessation temporally correlated with significant regressions in sleep electrophysiology and behavioral responsiveness, providing strong support for the hypothesis that CT-DBS modulates sleep electrophysiology via upregulation of daytime frontostriatal activation and system-level engagement. Modulation of sleep dynamics in response to diurnal neuronal activity has been well described in both animal and human studies. Across species, progressive wakefulness is associated with increased cortical excitability (38–42), increased neuronal firing rates (43, 44), and increased extrasynaptic glutamate levels (45). Subsequent non-REM sleep episodes are characterized by an initial maintenance of high neuronal firing rates, increased cortical synchrony, and upregulated slow wave activity in regions corresponding to increased neuronal activation during wakefulness (46–48). Successive non-REM sleep episodes show a progressive decline in each of these features (43). Accounting for these sleep-wake dynamics, growing evidence indicates that wakefulness creates a net increase in synaptic strengths that requires sleep processes for renormalization; a concept known as the “synaptic homeostasis hypothesis” (SHY) (49–51). Importantly, sustained high firing rates alone, such as those produced by CT-DBS, are insufficient to produce the changes in SWS observed here. Rather, SWS changes are more likely to result from system-level wakeful engagement with the environment that results in synaptic potentiation (38, 47, 52). The SHY therefore predicts that the marked improvements in sleep electrophysiology seen in our patient subject during CT-DBS reflect fundamental changes in synaptic potentiation occurring during wakefulness.

Further supporting this inference, daytime CT-DBS is known to upregulate the long-term potentiation (LTP)-related immediate early gene zif268 within neocortex (53). Similar gene expression patterns are expressed during periods of REM sleep following wakeful LTP (54); rodent studies have implicated these REM periods as instrumental in the consolidation of CT-DBS-induced learning (53). Accordingly, the selective appearance of REM sleep with CT-DBS in our patient subject likely reflects changes in LTP-related gene expression induced by the daily 12 h CT-DBS periods. Taken together, our findings suggest that the restoration of both non-REM sleep architecture and REM sleep episodes during CT-DBS may provide an indirect marker of meaningful daytime engagement across a range of sensory and associative processing systems within the forebrain.

Our finding that improvements in sleep electrophysiology are lost following withdrawal of CT-DBS suggests further that this process can be reversed. Specifically, sub-threshold wakeful activation may insufficiently engage organized neuronal dynamics needed for synaptic potentiation. Under-activated networks would therefore fail to produce the homeostatic sleep pressure necessary for large-scale neuronal synchronization and synaptic scaling during sleep. This general mechanism has precedence in the healthy brain. Following periods of arm immobilization, healthy individuals demonstrate localized wakeful synaptic depression and reductions in sleep slow wave activity over contralateral sensorimotor cortex (55). In the deafferented brain, the reduction of thalamocortical outflow associated with CT-DBS discontinuation would be expected to result in decreased cortical activation and synaptic depression, culminating in a progressive loss of wakeful frontocortical excitability and diminished homeostatic sleep-wake processes over time. The reduced behavioral responsiveness and degradation of organized sleep architecture after CT-DBS withdrawal at TP5 supports this inference. Collectively, these observations raise the possibility that restoration of synaptic homeostasis during sleep may be a process that is re-engaged in the severely injured brain only after sufficient increases in large-scale organized neuronal firing patterns emerge across the cerebral cortex to produce a net increase in synaptic strength during wakefulness. Such reinstatement of large-scale network engagement, including both glutamatergic synaptic potentiation and GABAergic firing rates (56), provides a testable mechanism for the observed changes in sleep architecture with CT-DBS.

As an exception to the observed global regressions following CT-DBS discontinuation, channel C3 displayed retained sleep spindles and improvements in SWS delta power at TP5 (Figures 3B,C), as well as the emergence of high frequency beta and healthy “Mu” or “wicket” rhythms (~8–13Hz) during wake (57, 58) (Figure 3A). Of note, although this patient subject was unable to communicate, he retained a high-level of emotional responsiveness consistent with his sense of humor prior to the injury. These unique dynamics underscore the structural preservation of the patient subject's left temporal cortex (Supplementary Figure 1) as well as verify the functional preservation of his left temporal language processing capabilities. Mechanistically, continued improvements in cortical regions underlying C3 may have resulted from local restructuring as the result of restored neuronal activation across relatively preserved cortical substrate during CT-DBS. Such changes would not be unprecedented; Thengone et al. (1) recently demonstrated prominent changes in structural connectivity emanating from Broca's area following implementation of assistive communication technology in a minimally conscious state patient. This independent EEG pattern exhibited by a localized brain region in our patient subject underscores the impact that upregulated neuronal activation can have on the recovery of functional circuitry in structurally intact brain regions.

### An Underactive Prefrontal Cortex may Permit Ventral Limbic Over-activation, Resulting in Alpha-Delta Sleep

Perhaps the most novel and interesting finding observed here is the mixing of alpha and delta rhythms during sleep, originally reported by Adams et al. (15) and identified here as alpha-delta sleep (16). Although the functional role and underlying circuit mechanisms of alpha-delta sleep have remained elusive (59, 60), the phenomenon has been reported in a variety of conditions including fibromyalgia/chronic fatigue (25, 26), rheumatoid arthritis (24), schizophrenia (16), major depressive disorder (29) with implications for suicidality (61), anxiety (28), and in healthy individuals with induced pain and/or arousal during sleep (62). To our knowledge, this is the first report of alpha-delta sleep in the severely injured brain. The persistence of the alpha-delta sleep phenotype across a range of neurological conditions, and now severe brain injury, invites us to consider a common underlying mechanism.

**Figure 6:**
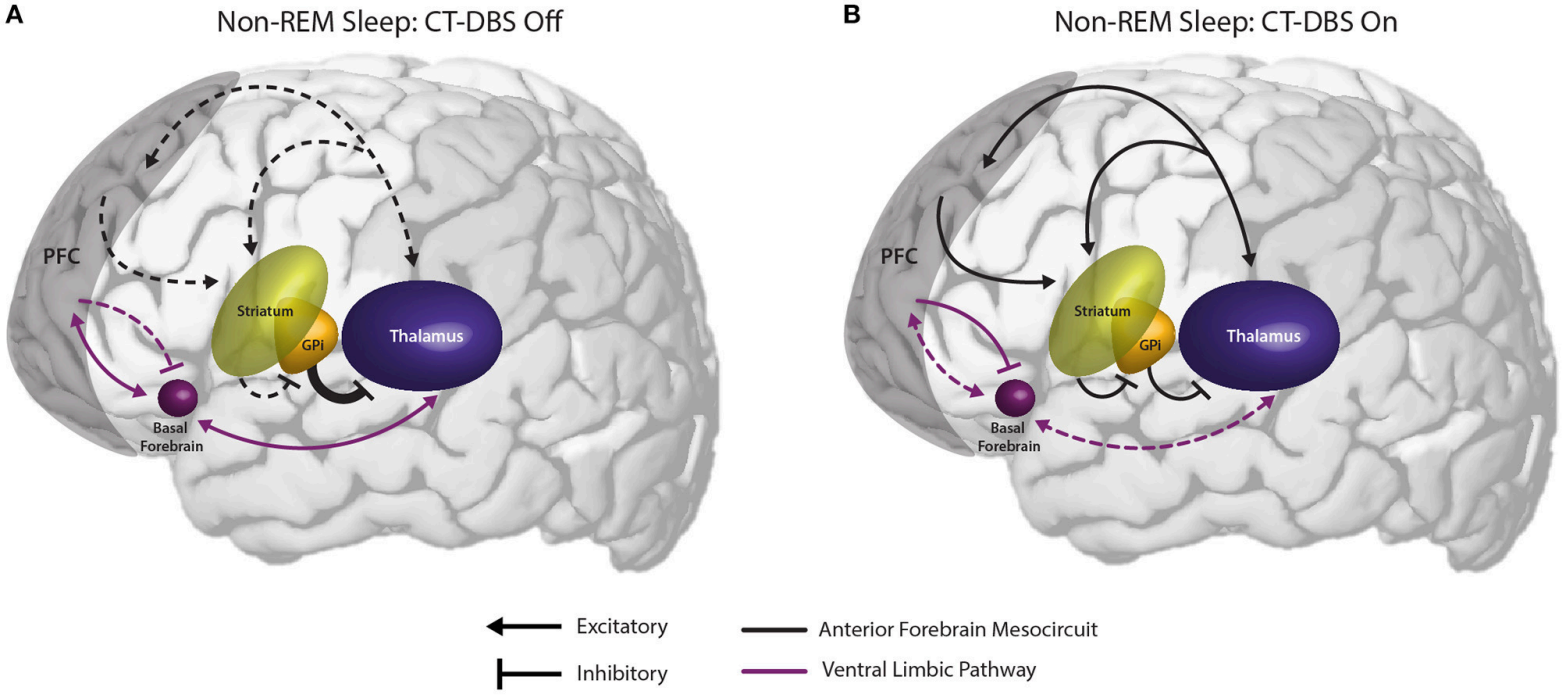
Proposed mechanism linking the observed effects of CT-DBS in our patient subject with anterior forebrain mesocircuit function and the generation of alpha-delta sleep. **(A)** Anterior forebrain mesocircuit dysfunction in disorders of consciousness. In severe brain injury, widespread deafferentation results in functional downregulation of the prefrontal cortex (PFC) via functional disfacilitation and structural deafferentation of central thalamic neuronal populations (30). Under these conditions, medium spiny neurons of the striatum fail to reach firing threshold, resulting in released inhibition of the globus pallidus interna (GPi). Excess firing of the GPi is proposed to result in additional inhibition of central thalamic components, preventing sufficient thalamocortical output, and tilting the excitatory/inhibitory balance needed for the consistent maintenance of consciousness [see Schiff 30]. Such reduced activation of the PFC during wake may result in insufficient accumulation of homeostatic sleep pressure and correspondingly under-activated prefrontal GABAergic networks during sleep. Failure to inhibit ventral limbic structures such as the basal forebrain would result in excess activation of thalamocortical cells and the intrusion of alpha oscillations during SWS. **(B)** Restoration of mesocircuit function and alpha-delta sleep alleviation during CT-DBS. CT-DBS drives thalamocortical output, resulting in restored wakeful excitation of the PFC. Increased PFC engagement during wakefulness produces an accumulation of homeostatic sleep pressure, facilitating the inhibition of ventral limbic structures during sleep via upregulation of SWS-producing GABAergic interneurons. Without excess thalamic activation by ventral structures during SWS, depolarization necessary for alpha-producing high threshold thalamocortical bursting is not achieved and alpha-delta sleep does not occur. Image adapted from The Allen Human Brain Atlas. ©2010 Allen Institute for Brain Science. Allen Human Brain Atlas. Available from: human.brain-map.org.

We observe that the conditions in which alpha-delta sleep is reported fall into two mechanistic categories: (1) those characterized by a primary pathology of cerebral hypofrontality or (2) those characterized by a primary upregulation of ventral limbic activation. Both mechanisms result in an increase in limbic system activity during sleep, either via under-activation of the descending corticothalamic pathway needed to drive homeostatic sleep pressure or an overactivation of the ascending pathways that maintain wakefulness. Accordingly, we hypothesize that the appearance of alpha-delta sleep is indicative of a failure of the prefrontal cortex to sufficiently inhibit excitatory output from ventral structures to the thalamus during the shift into synchronized cortical activity for SWS (Figure 6). Specifically, we identify the basal forebrain as a likely generator of thalamic depolarization in alpha-delta sleep due to its cholinergic projections to the thalamus (63–65). Support for this mechanism is provided by simulation and *in vitro* studies of both alpha oscillations (66–69) and alpha-delta sleep expression (70). Regarding alpha production by basal forebrain cholinergic projections, the activation of muscarinic acetylcholine receptors on reticular nuclei, thalamocortical, and high-threshold thalamocortical cells, as well as on somatosensory and visual thalamic nuclei, has been evidenced to produce alpha oscillations in thalamic models (69) and cat *in vitro* slice recordings (67), respectively. Conversely, follow-up *in vitro* studies show that direct thalamic application of a muscarinic acetylcholine receptor antagonist reduces high-threshold thalamocortical cell bursting, and in turn thalamic and cortical alpha power (68). Additional simulation studies demonstrate that alpha-delta sleep generation may originate in aberrant thalamic depolarization during SWS, specifically of “high-threshold” thalamocortical cells that serve as the putative generators of awake alpha (70).

Critically, the novel finding that alpha-delta sleep is modulated by CT-DBS lends strong support to the validity of this prefrontal-ventral dysfunction model of alpha-delta sleep generation. In our patient subject, CT-DBS restored bulk activation of the frontal cortices during the day, likely facilitating the reinstatement of top-down limbic inhibition (56) and driving activity-dependent increases in homeostatic sleep pressure. This increase in frontocortical GABAergic tone would be expected to carry over into sleep via sustained alterations of GABAergic firing rates (43) and the mutual reinstatement of synchronous cortical slow wave activity needed for synaptic scaling [see Allada et al. (71) for a detailed review]. With proper inhibition of ventral limbic structures by the frontal cortex during SWS, there would be minimal excess corticothalamic excitation and therefore attenuation of the alpha-delta sleep phenotype (69). In our patient subject, when CT-DBS was eventually discontinued, daytime frontocortical network activation was reduced, likely resulting in a gradual lifting of frontal inhibition over limbic structures during SWS and the observed re-emergence of alpha-delta sleep.

### Restoring Frontocortical GABAergic Tone Reduces Alpha-Delta Sleep

The prefrontal-ventral dysfunction model of alpha-delta sleep provides a consistent mechanism across several conditions in which alpha-delta sleep has been documented. Patients with fibromyalgia demonstrate reduced gray matter volume of the frontal cortex alongside increased structural connectivity of the amygdala (72, 73). Schizophrenia is associated with prefrontal GABAergic deficits and thalamocortical hypoconnectivity (74–76). Major depression is characterized by prefrontal gray matter reductions (77) and GABAergic interneuron deficits (78, 79), as well as ventral hypermetabolism that persists from wakefulness into non-REM sleep (80). Individuals with anxiety and PTSD demonstrate reduced prefrontal regulation of the amygdala (81–83), which is present in both fear conditions and resting states (84, 85); The high prevalence of sleep disturbances in PTSD suggests that prefrontal-amygdala dysfunction persists into sleep states as well. Together, these commonalities suggest that alpha-delta sleep dynamics may be indicative of the presence and/or severity of prefrontal-ventral dysregulation across behavioral diagnoses.

In support of the generalizability of this model, the alleviation of frontal GABAergic deficits by gamma hydroxybutyrate (GHB/sodium oxybate) has been found to suppress alpha-delta sleep in several conditions (86–90). GHB is an activity-dependent neurotransmitter synthesized within GABAergic interneurons [reviewed in detail by Maitre et al. 91]. Importantly, these GHB-containing interneurons play a critical role in the endogenous regulation of sleep-wake cycles by inhibiting cholinergic structures such as the basal forebrain (92). At increased doses used for exogenous administration, GHB exerts inhibitory effects by directly binding GABA-B receptors (93, 94); a necessary step for the homeostatic modulation of firing rates (95). Accordingly, the demonstrated suppression of alpha-delta sleep and dose-dependent upregulation of SWS by GHB likely occurs through a GABA-B receptor-mediated process analogous to both the endogenous production of homeostatic sleep pressure through normal wakeful activity and the exogenous driving of organized frontocortical networks with CT-DBS. Kothare et al. (96) reported a case of sodium oxybate use in an 8-year-old boy with a prior disorder of consciousness produced by encephalitis at age four, providing strong support for this mechanism. The boy presented with disseminated encephalomyelitis with thalamic lesions, poor sleep efficacy, and alpha-delta sleep alongside severe cognitive and attentional regulation problems indicating prefrontal downregulation characteristic of anterior forebrain mesocircuit dysfunction (30). Following 6 months of sodium oxybate treatment beginning 4 years after the initial event, he showed improvements in all measures of sleep including increased SWS and the disappearance of alpha-delta sleep, as well as improvements in measures of attention, executive function, and impulse control. We suggest that these findings, in concert with those of our patient subject, complimentarily underscore the bi-directional carry-over effects of GABAergic upregulation between sleep and wakeful states. In our patient subject, upregulation of frontocortical GABAergic circuits during wakefulness resulted in the recovery of sleep architecture; In Kothare et al.'s patient subject, upregulation of GABAergic circuits during sleep resulted in the recovery of wakeful frontocortical function. Accordingly, we emphasize the notion that state-dependent activation of GABAergic circuits exerts a 24 h cyclical influence over organized neuronal function.

### Limitations and Future Directions

The present study has important limitations to consider. Due to the single-patient, observational nature of this study, interpretations of causality must be made with caution. Nevertheless, our primary findings of regression in both behavioral responsiveness and sleep features are temporally correlated with the discontinuation of CT-DBS ~12 months prior. As there had been no changes in medication or rehabilitation that could account for the sudden shift in these features, we feel it is reasonable to attribute these changes to a shift in neuronal dynamics resulting from the withdrawal of CT-DBS.

Furthermore, it is possible that our findings and proposed model will not generalize to the larger population of individuals with disorders of consciousness. However, evidence indicates that the prevailing network dynamics observed in this patient subject are not unique, but instead mechanistically characteristic of the severely deafferented brain (97). Larger studies of sleep dynamics in patients with disorders of consciousness are needed to further clarify the potential value of sleep electrophysiology as a meaningful indicator of wakeful brain function. Additionally, although this is the first case in which alpha-delta sleep has been characterized in a patient with severe traumatic brain injury, we have previously described this phenomenon in a small number of minimally conscious state patients (98). Accordingly, we suggest that alpha-delta sleep and the implicated network dynamics may be present across many more patient subjects. Future studies should seek to identify the prevalence of alpha-delta sleep in individuals with disorders of consciousness, as well as to experimentally investigate the described prefrontal-ventral dysfunction model as a mechanism of alpha-delta sleep generation across populations. We suggest that our proposed model provides several possible experimental evaluations to determine its predictive validity in populations with alpha-delta sleep.

## Author Contributions

JG: Study concept and design, data acquisition, data analysis and interpretation, manuscript preparation and revision; ZA: Data acquisition, data analysis, manuscript revision; PF: Clinical assessment, manuscript revision; NS: Study concept and design, data acquisition, clinical assessment, data interpretation, manuscript preparation and revision.

### Conflict of Interest Statement

The authors declare that the research was conducted in the absence of any commercial or financial relationships that could be construed as a potential conflict of interest.
