# Supplementary material for: Daytime Central Thalamic Deep Brain Stimulation Modulates Sleep Dynamics in the Severely Injured Brain: Mechanistic Insights and a Novel Framework for Alpha-Delta Sleep Generation
Source: Front Neurol. 2019 Feb 4;10:20. doi: 10.3389/fneur.2019.00020 (PMC6369150; doi:10.3389/fneur.2019.00020)
Supplement: Supplementary file 1 [file Data_Sheet_1.docx]

Supplementary Material

**Daytime Central Thalamic Deep Brain Stimulation Modulates Sleep Dynamics in the Severely Injured Brain: Mechanistic Insights and a Novel Framework for Alpha-Delta Sleep Generation**

Jackie L. Gottshall*, Zoe M. Adams, Peter B. Forgacs, Nicholas D. Schiff

*** Correspondence:** Jackie L. Gottshall: [jag2037@med.cornell.edu](mailto:jag2037@med.cornell.edu)

# Supplementary Figures

**Supplementary Figure 1.** Four representative T1 weighted horizontal brain images illustrating left greater than right atrophy and ventricular dilatation secondary to diffuse axonal injury. Note relative preservation of left parietal cortical volume in comparison to remaining left hemisphere cortical regions (corresponding to cortex under electrode C3).
